# Pathologic Fracture as Presentation of Generalized Lymphangiomatosis in an Eight-Year-Old: A Case Report and Literature Review

**DOI:** 10.7759/cureus.24380

**Published:** 2022-04-22

**Authors:** Alex R Ghorishi, Nicole J Levin, Kranthi Nomula, Jared Green, Eric Eisner

**Affiliations:** 1 Medicine, Florida Atlantic University Charles E. Schmidt College of Medicine, Boca Raton, USA; 2 Pediatrics, Joe DiMaggio Children’s Hospital, Hollywood, USA; 3 Interventional Radiology, Memorial Healthcare, Hollywood, USA; 4 Orthopedic Surgery, Memorial Healthcare, Hollywood, USA

**Keywords:** lymphatics, pathologic fracture, generalized lymphangiomatosis, radiology, pediatrics

## Abstract

Pathologic fractures commonly occur secondary to abnormal skeletal physiology in the context of benign or malignant lesions. Rarely, pathologic fractures may occur in the context of a lymphatic abnormality, such as generalized lymphangiomatosis. This rare disorder is characterized by variable presentations in a broad age range of patients. By understanding the effect of widespread lymphatic anomalies on various organ systems, clinicians will be able to make this diagnosis earlier and with more certainty.

## Introduction

Generalized lymphatic anomaly (GLA), previously known as generalized lymphangiomatosis, is a rare congenital disease characterized by aberrant proliferation of lymphatic vessels in bone and soft tissue, which typically affects children or young adults. Diagnosis can be delayed because of the rarity of the condition, increasing morbidity and mortality. Having high clinical suspicion for GLA in the setting of widespread osteolytic lesions and cystic lymphatic lesions in soft tissue may enable earlier diagnosis and prevent the morbidity and mortality associated with the condition.

GLA is a nonneoplastic congenital condition in which lymphatic vessels proliferate in an unregulated manner, causing the formation of dilated and thin-walled cystic structures. Although single organ involvement is possible, the condition more commonly affects multiple organ systems, typically the lungs and bone. GLA most commonly presents in the first two decades of life. The incidence and clinical features of GLA are unknown, challenging even the most seasoned clinicians. A multidisciplinary team of pediatricians, orthopedists, and radiologists is needed to diagnose and manage patients with this disease [1,2].

A previous case series of 53 patients with GLA demonstrated that 49% presented with pleural effusion, 45% with pulmonary infiltrates, 39% with bone lesions, 19% with splenic lesions, 15% with cervical involvement, 9% with disseminated intravascular coagulation, and 7% with skin involvement [3]. When diagnosed in children, GLA carries a poor prognosis with a mortality rate of 39% in patients less than 16 years of age. In adults, the mortality approaches 0% [3].

Once patients present with symptoms such as respiratory distress, abdominal pain, or, in this case, pathologic fracture, initial imaging may provide clues toward the diagnosis. Experienced radiologists can identify the classical imaging features, including radiolucent bone lesions on plain radiographic imaging [4]. Other findings on chest X-rays include chylous pleural effusion, soft tissue masses, and widening of the mediastinum [5]. Once these features are recognized, whole-body magnetic resonance imaging (MRI) is necessary to determine the extent of organ involvement [1]. MRI will reveal well-demarcated osteolytic bone lesions with sclerotic borders that can affect any bone [1]. Extraosseous involvement can manifest as multicystic non-enhancing masses with microlobulated margins and internal septation in soft tissue and parenchymal organs [6]. Diagnosis can be made solely based on the history, physical examination, and findings on imaging. Without treatment, the expansion of these lytic bone lesions and cystic lymphatic masses can lead to morbidity and mortality from respiratory distress and hemodynamic instability due to mass effects in the thoracic cavity.

## Case presentation

An eight-year-old male with a history of attention deficit hyperactivity disorder presented to an orthopedic office with an injury to his right forearm and elbow after falling onto his outstretched arm while dancing. His mother suspected that he had broken his arm due to intractable pain. Subsequent X-ray of the right forearm and right humerus in anteroposterior and lateral views not only confirmed a fracture in the proximal right radial diaphysis but also revealed an underlying lytic lesion in the humerus concerning osteosarcoma. MRI was performed in the outpatient setting to investigate the underlying cause of the pathologic fracture, which showed findings concerning osteosarcoma. The patient was then referred to the emergency department for further workup.

In the emergency department, a comprehensive review of symptoms was conducted. Pertinent negatives included a lack of constitutional symptoms (fever, chills, fatigue, weakness, and weight loss) and no respiratory distress, abdominal pain, nausea, vomiting, skin lesions, adenopathy, or easy bruising. The only significant positive finding in the history was arthralgias, not only in the injured arm but also in the left knee. Physical examination demonstrated an active, well-appearing eight-year-old male in no acute distress. His physical examination was only remarkable for diffuse tenderness overlying the patella and throughout the right arm.

**Figure 1:**
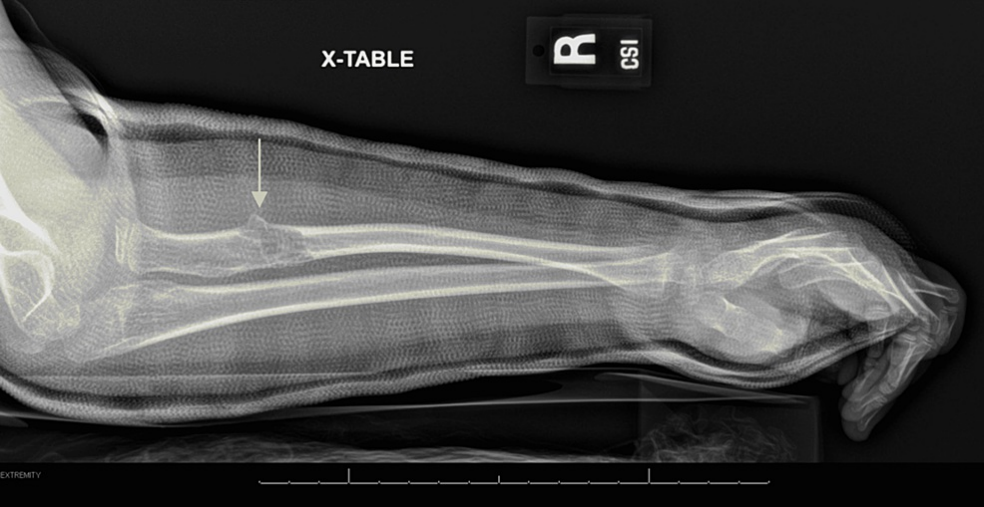
Pathologic fracture of the proximal right radial diaphysis with underlying lytic lesions (arrow).

An X-ray skeletal survey was ordered, which revealed multiple lytic lesions in the right humerus, as seen in Figure 1, as well as in the right fourth rib, left sixth rib, calvarium, mandible, left iliac wing, right proximal femur and distal diaphysis, left proximal femur and mid diaphysis, and left proximal tibia metaphysis. Accompanying these bony lesions was a mediastinal mass, obscuring the left heart border but with no significant airway compression or associated pleural effusions.

**Figure 2:**
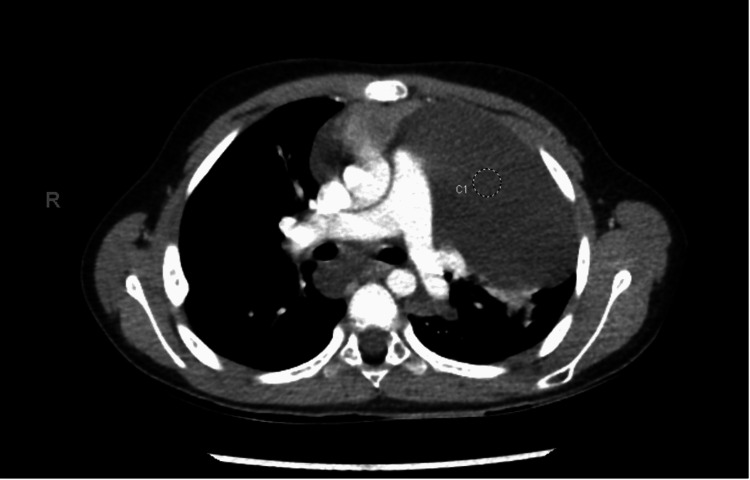
Chest CT demonstrating large infiltrative multicompartmental mediastinal mass with cervical extension on the left and inferior extension to the esophageal hiatus, encasing mediastinal structures.

Due to the concerning mediastinal mass, CT scans of the abdomen, chest, pelvis, and neck with contrast were conducted. The results of the CT scan in Figure 2 demonstrated a large, infiltrative hypodense mass involving all compartments of the mediastinum on the left. The mass extended superiorly to the cervical region on the left to the level of the C5 vertebra, just above the superior aspect of the thyroid, and bilaterally to the inferior aspect of the thyroid gland. The inferior border extended posteriorly to the level of the esophageal hiatus. The mass measured at least 13 cm in the transverse dimension by 11 cm in the anterior-posterior dimension by 17 cm in the craniocaudal dimension. Although the great vessels were diffusely encased, no significant mass effect was observed. Further, the trachea and mainstem bronchi were involved but without significant narrowing, except for the left distal mainstem bronchus. The esophagus and descending thoracic aorta were also encased by the mass. Although no pleural or pericardial effusions were appreciated, areas of consolidation in the left lower lobe were present. The mass not only caused narrowing of the distal left mainstem bronchus and lobar branches but also diffuse atelectasis in the left upper lobe and lingula. Other pulmonary findings included ground-glass attenuation throughout the left lung with air trapping in the anterior left lower lobe and lingula. No enlarged axillary, mediastinal, or hilar lymph nodes were appreciated, lowering the possibility of metastatic malignant disease. In addition to innumerable osseous lytic lesions, multiple hypoenhancing splenic lesions measuring up to 1.4 cm were found. The constellation of findings was alarming for the possibility of a diagnosis of generalized lymphatic anomaly. However, further evaluation of the mediastinal mass for internal complexity via MRI was warranted to exclude malignant disease.

Due to the concerning large mediastinal mass, with possible mass effect on adjacent airways and great vessels, the patient was transferred to the ICU for close monitoring. He remained afebrile and hemodynamically stable, with 100% oxygen saturation on room air. His range of motion, sensation, and strength remained fully intact, and no edema was appreciated on the physical examination. After a couple of uneventful days in the ICU, the decision was made to transfer the patient back to the general pediatric unit.

**Figure 3:**
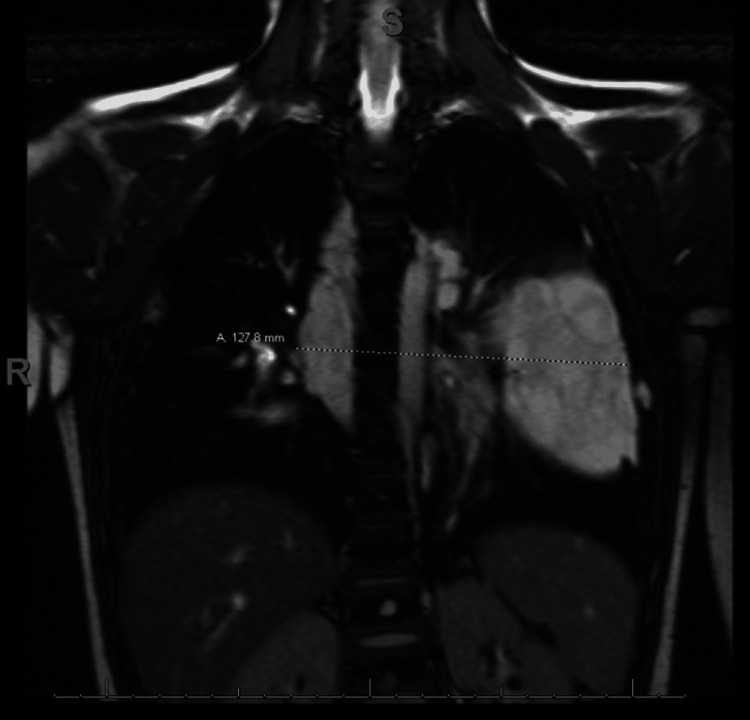
MRI demonstrating macrocystic infiltrative multicompartmental mediastinal mass measuring 13 × 12 × 17 cm suggestive of a lymphatic malformation.

Unfortunately, due to motion, only limited sequences of the chest could be obtained on magnetic resonance imaging. The MRI results seen in Figure 3 revealed no internal mass-like enhancement or restricted diffusion. These results were concurrent with the results from CT, suggesting a high likelihood of lymphatic malformation, a typical finding in GLA.

Although diagnostic imaging findings strongly suggested the diagnosis of GLA, a definitive diagnosis was obtained via ultrasound-guided percutaneous drainage of the anterior mediastinal mass and CT-guided bone biopsy of a cystic lesion in the pelvic bone. The dominant locule of the cystic mediastinal mass was aspirated with a 5-Fr Yueh needle via ultrasound guidance, yielding approximately 400 mL of serous, non-bloody fluid. The cyst did not reaccumulate fluid post-drainage. Doxycycline sclerotherapy was performed subsequently using 200 mg doxycycline in 10 mL water.

Next, a lesion in the left iliac bone was biopsied under ultrasound and CT guidance. The lesion was filled with low-viscosity serosanguinous fluid, which could not be sampled via core needle biopsy. Thus, two cores were sampled from the adjacent bone. The procedure was performed with no complications under general anesthesia.

Flow cytometry of the mediastinal cyst fluid revealed no monoclonal B-cell population or phenotypically abnormal T-cell population. Surgical pathology of the bone specimen revealed rare lymphatic channels, with endothelial cells immunopositive for D2-40 and seams of woven bone as opposed to lamellar bone. The histopathology of the bone sample was consistent with GLA. These findings ruled out leukemia, lymphoma, and the more aggressive lymphatic defect known as kaposiform lymphangiomatosis (KLA).

## Discussion

Abnormal development of the lymphatic system can manifest as rare benign neoplasms known as lymphangioma. When these become widespread, the condition becomes known as a generalized lymphatic anomaly. Due to the rarity of the condition, the clinical features are not completely characterized, and the cause and prognosis of the disease are poorly understood. Prior case reports in patients with suspected GLA have reported involvement of the bones, mediastinum, spleen, liver, lungs, neck, and pleura. The prevalence and incidence of the disease are likely underreported due to the asymptomatic presentation in many patients. If symptomatic, typical complaints include difficulty breathing, abdominal pain, ascites, and other symptoms due to the compressive effect of the large cystic mediastinal masses [7]. Theoretically, any organ can be involved, except the central nervous system, due to its lack of any lymphatic structures [8].

Although a classic lymphangiomatosis patient will present emergently with complaints of respiratory distress and fluid backup, pathologic fractures due to lytic bone lesions are possible. Thus, GLA should be considered in the differential diagnosis in pediatric patients with pathologic fractures. Pathologic fractures in children may result from a wide variety of pathologic processes, including metabolic diseases, infections, and tumors. Various benign bone tumors such as unicameral bone cysts, aneurysmal bone cysts, non-ossifying fibromas, and fibrous dysplasia may weaken the structural integrity of bone and cause fractures. However, malignant processes such as metastases from neuroblastoma and Wilms tumor, leukemia, Ewing’s sarcoma, and fibrosarcoma can predispose to unexpected fractures [9]. Thus, a thorough history, physical examination, and review of plain radiographs are of utmost importance in distinguishing benign versus malignant causes of pathologic fracture. In patients with multifocal lytic bone lesions in the absence of signs or symptoms of leukemia or lymphoma, a high clinical index of suspicion for GLA is warranted. Increasing awareness about GLA is crucial to the early diagnosis and treatment of these patients. Delayed diagnosis of GLA is common due to poor knowledge of this condition among radiologists and clinicians. Once a generalized lymphatic abnormality is suspected, clinicians must rule out a distinct lymphatic anomaly known as kaposiform lymphangiomatosis (KLA), a subtype of GLA according to the 2018 updated International Society for the Study of Vascular Anomalies (ISSVA) classification [10]. Another similar entity known as Gorham-Stout disease (GSD) should also be ruled out.

GLA can be differentiated from KLA and GSD via histopathologic and radiographic findings. Microscopically, GLA is characterized by patterns of anastomotic lymphatic channels with thin walls lined by flattened endothelial cells. However, spindled endothelial cells in the background of the malformed lymphatic channels are more consistent with the diagnosis of KLA. The histological hallmark of KLA is clusters or sheets of hemosiderotic spindled endothelial cells aligned in parallel in the interstitium within the context of abnormal and dilated lymphatic channels [11]. The radiological findings in the osteolytic lesions of GLA/KLA differ from GSD. In GLA/KLA, multiple lytic bone lesions are typically noted but without progression or invasion past the medullary cavity. In GSD, bony lesions tend to progress over time, infiltrating the surrounding bone and causing loss of cortical bone [11].

Clinical features can also be used to distinguish GLA from KLA and GSD. While GLA and KLA tend to have multiple organ involvement (mediastinum, lungs, bone, and spleen) causing pleural and pericardial effusion, ascites, and lymphedema, GSD causes discrete osteolysis of any bone that inevitably progresses, leading to the catastrophic destruction of the bone. Thus, pathologic fracture is much more common in GSD but cannot exclude GLA. However, other findings such as joint abnormalities, leg length discrepancy, asymmetric girth, and scoliosis are far more suggestive of GSD than GLA [11]. Further, pain and swelling due to destructive osteolytic lesions are more common in GSD than in GLA. GLA and KLA patients typically have more frequent vertebral involvement than GSD patients, with the lumbar spine at high risk [12].

Findings that may raise suspicion for KLA as opposed to GLA include the presence of hemorrhagic effusion, extensive retroperitoneal involvement, a deteriorating clinical course, or associated hematologic abnormalities. Most patients with KLA present in childhood with respiratory or bleeding concerns due to thrombocytopenia, hypofibrinogenemia, and prolonged PT/aPTT associated with the condition. The common manifestations of pathologic bleeding in KLA patients include epistaxis, scleral hemorrhage, ecchymosis, vaginal bleeding, epidural hematoma, and hemorrhagic effusions. Video-assisted thoracoscopy of KLA patients demonstrated hemorrhagic plaques on the visceral pleura associated with dilated tortuous blood vessels. Ultimately, a biopsy is necessary for differentiating these different clinical entities [11].

Treatment in patients with GLA is mostly symptom-based. For example, for the patient described in this report, sclerotherapy was used to prevent refilling of the mediastinal mass, which could have led to respiratory distress, ascites, and obstructive shock by compressing the vena cava. The sclerotherapy may even reduce the chances of developing pleural and pericardial effusions. If these types of effusions did develop, pleural fluid drainage or pleurodesis would be warranted. Other options for palliative care include radiotherapy, interferon-alpha, steroids, and other chemotherapeutic drugs [13]. Beta-blockers such as propranolol can also be used, and these agents offer the benefit of significantly fewer side effects compared to radiotherapy and chemotherapy [14].

## Conclusions

GLA is a potentially life-threatening congenital disease characterized by the formation of aberrant lymphatic channels, which are widely distributed in bone and soft tissue throughout the body. Due to its rare nature and the severity of its prognosis, practitioners should be familiar with its presentation and characteristic findings. Early diagnosis and treatment may improve outcomes for these patients. However, more research is needed to optimize therapeutic interventions for these patients.
